# Do Synthetic Fragrances in Personal Care and Household Products Impact Indoor Air Quality and Pose Health Risks?

**DOI:** 10.3390/jox13010010

**Published:** 2023-03-01

**Authors:** Gandhi Rádis-Baptista

**Affiliations:** 1 School of Pharmacy, Dentistry and Nursing, Federal University of Ceará, Fortaleza 60440, CE, Brazil; gandhi.radis@ufc.com; Tel.: +55-85-996-886-054; 2 Laboratory of Biochemistry and Biotechnology, Institute for Marine Sciences, Federal University of Ceará, Fortaleza 60440, CE, Brazil

**Keywords:** fragrance chemicals, synthetic fragrances, personal care and household products, volatile organic compounds, indoor air quality, olfactory receptors, endocrine disruptor chemicals

## Abstract

Fragrance compounds (synthetic fragrances or natural essential oils) comprise formulations of specific combinations of individual materials or mixtures. Natural or synthetic scents are core constituents of personal care and household products (PCHPs) that impart attractiveness to the olfactory perception and disguise the unpleasant odor of the formula components of PCHPs. Fragrance chemicals have beneficial properties that allow their use in aromatherapy. However, because fragrances and formula constituents of PCHPs are volatile organic compounds (VOCs), vulnerable populations are exposed daily to variable indoor concentrations of these chemicals. Fragrance molecules may trigger various acute and chronic pathological conditions because of repetitive human exposure to indoor environments at home and workplaces. The negative impact of fragrance chemicals on human health includes cutaneous, respiratory, and systemic effects (e.g., headaches, asthma attacks, breathing difficulties, cardiovascular and neurological problems) and distress in workplaces. Pathologies related to synthetic perfumes are associated with allergic reactions (e.g., cutaneous and pulmonary hypersensitivity) and potentially with the perturbation of the endocrine-immune-neural axis. The present review aims to critically call attention to odorant VOCs, particularly synthetic fragrances and associated formula components of PCHPs, potentially impacting indoor air quality and negatively affecting human health.

## 1. Introduction

Pharmaceutical (drug) pollution is becoming a recognized ecological and environmental concern, especially in different water systems [1,2,3,4]. Pharmaceuticals and personal care products (PPCPS) that range from prescription and over-the-counter drugs to cosmeceuticals and formula components appear as potential environmental contaminants collectively named “emerging pollutants”. Personal care products (PCPs) are formulated with several classes of organic chemicals (e.g., fragrances, antiseptics, additives, fixatives, preservatives, and solvents) that can enter all environmental compartments and pose considerable risks to human health and marine and terrestrial wildlife. These ingredients include: alkylphenols, antimicrobials, bisphenols, cyclosiloxanes, ethanolamines, parabens, phthalates, and benzophenone to mention a few classes of hundreds of chemicals. They are eventually persistent, cumulative, and transformable [5]. These and other emerging chemicals and chemicals of concern have been detected in indoor air as the detection techniques are becoming more comprehensive and sensitive. Moreover, analytical methods, combined with human biomonitoring, can precisely ascertain these chemicals’ contamination and harm levels in the indoor environment [6]. Worthy of note, VOCs, in the form of compressed aerosols, which are propellants of personal care and household products (PCHPs) like air fresheners, colognes, and perfumes, body and hair sprays, cleaners, among others, accounted for astonished global emissions of over 1 Tg per year in 2018 and could surpass 2 Tg per year in 2050, representing a negative impact on the atmosphere and human health [7].

Many natural and synthetic fragrances are components of cosmetics and household products aiming to impart pleasant aromas (fragrances and perfumes) in the product formulation, such as body lotions, shampoos, soaps, laundry detergents, air fresheners, disinfectants, and vaporizers. Essential oils and fragrances of natural origin that are volatile and reactive chemical species contained in PCHPs can negatively impact indoor air quality due to, among other things, emissions of secondary pollutants such as formaldehyde [8]. Synthetic scents, primarily derived from petroleum, are of great concern because they are the cheapest, present in abundance, and consequently one of the main contributors to decreasing indoor air quality and increasing personal exposure and potential health risks. Synthetic fragrances include derivates of several chemical structures and organic functions: acids, alcohols, esters, aldehydes, acetals, nitrogen heterocycles, oximes, amides, amines, nitriles, Schiff base, oxygen heterocyclics, lactones, coumarins, ethers, sulfur heterocycles, thiols, sulfides, thiocyanates, dithiazides, and hydrocarbons. For instance, the transformation of alkenes, alkynes, dienes, and enynes results in carbo- and heterocyclic fragrances with natural and tunable scents [9].

The development and advances over the past two decades in odorant chemistry regarding the design, chemical synthesis, and their use in the modern fragrance (and flavor) industry comprise the content of a recent review [10]. For instance, synthetic terpenes and terpenoids constitute a well-succeeded class of artificial synthetic fragrances that mimic natural ones [11]; terpene-based fragrances (natural or synthetic) in indoor air are one of the predominantly emitted scents. However, terpenes in low concentration are usually not harmful to human health but are the secondary products of their reaction with ozone and the hydroxyl radical that generate, for example, formaldehyde and ultrafine particles [12]. Over six thousand organic chemicals are known as fragrance ingredients for PCHPs, and they have been clustered into chemical class-based functional groups, of which over two thousand are used in fragranced formulations [13]. In PHCPs, the designations “fragrance” and “perfume” may comprise complex mixtures of dozen to hundreds of chemicals instead of a single odorant compound, and these potentially can, individually or in combination, elicit adverse effects on biological systems and human health [14,15]. The increased concentration of fragrances and fragranced-associated VOCs in the indoor air may cause adverse cutaneous, respiratory, and systemic effects such as headaches, asthma attacks, breathing difficulties, cardiovascular and neurological problems, mucosal irritation, and contact dermatitis, as well as distresses in workplaces and public places due to personal exposure to fragranced products [16].

In the context of indoor and outdoor air contamination, in addition to the vast amount of food and energy that contribute to atmospheric and environmental pollution, the human population can move tons of chemicals contained in PCHPs for hygiene, cleaning, grooming, and embellishment. The present review aims to consider and call attention to odorant VOCs, particularly synthetic fragrances, and associated formula components of PCHPs, that potentially impact indoor air quality and negatively affect human health.

## 2. Synthetic Fragrances in Personal Care and Household Products and Potential Adverse Effects

Thousands of fragranced products (perfumes, colognes, body soaps, hand washes, shampoos, facial/hand cleansers/creams, deodorants) and household articles (air fresheners, disinfectants, fragrance diffusers, laundry/dish detergents, scented candles, surface cleaners) are in use daily worldwide. Natural (essential oils) or synthetic scents are vital constituents of PHCPs to make them more attractive to the olfactory perception or disguise the unpleasant odor of the formulation components. Despite the success of the synthetic fragrance industry, which reduces the exploitation of natural resources due to the demand for essential oils, it is unsurprising that synthetic odorant substances contained in PCHPs may target several organs and trigger a particular biological response other than olfaction. Olfactory receptors (ORs) are transmembrane protein receptors expressed in the olfactory epithelium and sensory neurons that transduce signals after recognizing and binding to myriad odorant molecules. A repository to explore olfactory receptors, odors, odorants, and odorant–receptor interactions is accessible that contains information on ORs from humans (851 ORs) and mice (1215 ORs), in addition to almost a thousand OR-odorant pairs and thousands of odorant-related chemicals (~4000 odorant compounds) [17]. This database aims to facilitate the understanding of the dynamic interplay between olfactory receptors-odor/odorant molecules interaction regarding their physicochemical and pharmacokinetic properties. In humans, ORs are also expressed in cells and tissues other than sensory neurons. ORs distribute in tissues as diverse as the testis, lung, intestine, skin, heart, blood cells, and even cancer tissues. They are involved in the modulation of critical cellular processes like cell-cell recognition, migration, proliferation, the apoptotic cycle, and exocytosis [18]. Activation of subtypes of ORs in human macrophages induces inflammasome assembly and the secretion of IL-1β, exacerbating atherosclerosis. Triggering another subtype of ORs promotes the expansion of tumor-associated macrophages that depress the immune response and promote tumor progression [19]. 

In combination, rose ketones and odorant agonists α- and β-ionone trigger a higher increase in the total tumor burden leading to an augmented generation of metastases in vivo in mouse models of prostate cancer. The increased tumor aggressiveness effect is worse with the combination of α-ionone with β-ionone than with each molecule alone [20]. The estrogen activity and the dual action of odorant chemicals are observable in synthetic sandalwood compounds: these odorants, used in the perfume industry, activate ORs and human nuclear estrogen receptor (α-ER), affecting both neuronal signaling (odor perception) and the ER-dependent transcriptional of specific genes. The activation of OR and α-ER provided a direct functional link between the ORs’ response and endocrine/hormonal systems in humans [21]. Fragrance molecules may trigger various acute and chronic pathological conditions because of repetitive human exposure, particularly in vulnerable populations. Previous research has suggested that synthetic perfumes may potentially cause perturbation of the endocrine-immune-neural axis. Disease symptoms related to fragrance chemicals may include neural disturbances (e.g., headache, depression, and migraine), skin and airway hypersensitivity, breast cancer and polycystic ovary syndrome, gynecomastia, liver and thyroid toxicity, reproductive problems, and teratogenic toxicity effects [22]. 

A database recently released contains information about 153 fragrance chemicals in children’s products [23]. This database classified the fragrance chemicals in children’s products based on their chemical structures, sources, chemical origins, odor profiles, physicochemical properties, and predicted data on absorption, distribution, metabolism, excretion, and toxicity (ADMET). Due to the vulnerability of children at an early age exposed to VOCs, the fragrances in children’s products are pollutants of concern since they appear as potential carcinogens, endocrine disruptors, neurotoxicants, phytotoxins, and skin sensitizers [23]. In a study of 42 fragranced baby products, over six hundred emitted VOCs were detected, of which approximately one-third are potentially hazardous. These VOCs comprised different chemical classes but predominated the fragrance compounds limonene, α-pinene, linalool, β-myrcene β-pinene, and acetaldehyde (additive/fragrance), and the solvents (ethanol, acetone) [24].

Neurotoxicity and allergic reactions are acute adverse effects due to odorant VOCs observed in sensitive populations. Phthalates (e.g., dimethyl phthalate and diethyl phthalate), synthetic musks (e.g., tonalide, galaxolide), and the so-called sensitizers (e.g., citral, linalool, limonene, geraniol, citronellol, eugenol, farnesol, auraptene, osthole, umbelliferone) have been implicated in many neurotoxic issues. Neurotoxicity ranges from interference with neurotransmitter release and inhibition of neurotransmitter enzymes to dysregulation of intracellular signaling in neurons and downregulation of gene expression [25]. The effects occurring at molecular levels in response to (synthetic) fragrances result in developmental delay, poorer adaptive function (socialization, communication, motor skills), more significant hyperactivity and impulsivity in children, poorer behavior like attention, externalization, emotional control, aggression and depression, and brain degeneracy and propensity to develop Alzheimer’s Disease as for phthalates. Except for the first generation of synthetic musks (e.g., nitromusks), which causes central and peripheral nervous system deficiencies and, consequently, aggressiveness, irritability, and other neurologic symptoms, most sensitizers (terpenes, coumarins) exert neuroprotective, anticonvulsant, and analgesic effects [25]. However, the question is, what if these positive neuromodulatory effects observed in experimental models are sustained indoors for long periods of exposure? Xenobiotics can target several organs and systems in the human body and thus represent hazardous chemicals, causing endocrine dysregulation through estrogen receptors, nuclear receptors, and steroidal receptors, resulting in distinct chronic diseases [26]. Fragrance-related phthalates (e.g., dibutyl-, dipentyl-, benzyl butyl-, diphenyl-phtalates) and synthetic musks (e.g., musk ketone, musk xylene, musk ambrette, musk moskene, musk tibetene, galaxolide, traseolide) are recognized endocrine disruptors likewise a handful of components of PCHPs formulation [27,28].

Fragrance chemicals are responsible for causing allergic reactions (fragrance allergy) like contact dermatitis, as indicated by clinical diagnosis through patch testing [29]. Among the frequent sensitizers that cause contact dermatitis are linalool and limonene hydroperoxides, hydroxyisohexyl 3-cyclohexene carboxaldehyde, treemoss and oakmoss absolute, isoeugenol, cinnamyl alcohol, and cinnamal. Fragrance sensitizers also cause other less common allergic adverse reactions like immediate contact reactions (contact urticaria), photosensitivity, and respiratory disorders [30]. Allergic sensitization of the respiratory tract and asthma caused by exposure to fragrances is controversial since the cause-effect is considered by some researchers more methodological than factual, and the inhalation of fragrance sensitizers might not be a health risk concerning allergy [31,32], being their clinical symptoms considered psychosomatics [33]. However, as aforementioned, fragrances (natural or synthetic) are volatile and reactive chemical species, and the generation of secondary pollutants may follow the emission of PCHP constituents. The terpenes’ gas- and surface reactions with ozone and the hydroxyl radical produce secondary pollutants, like formaldehyde and ultrafine particles, that can persist for extended periods in indoor air [8,12,34]. Despite the formation of secondary pollutants due to the terpene-ozone reaction and their harmful effects, the increased levels of inflammatory biomarkers in vitro and inflammatory response in the respiratory tract in vivo were admittedly of minor significance and relevance [12]. Even so, mixtures of odorant VOCs and fragrance chemicals in indoor air reach concentrations that usually exceed the outdoor, and people spend more time exposing themselves to contaminants indoors for long periods. In these conditions, the susceptible population experience exposure to mixtures of VOCs that surpass the base guidelines of low health risks [35]. The co-exposure to combinations (cocktails) of fragrance VOCs facilitates the sensitization and the acute and chronic adverse effects exerted by fragrances and components of PCHPs formulation [36,37]. Table 1 indicates some significant classes of fragrance chemicals and PCHPs’ formula components and associated adverse health effects mentioned in this article.

## 3. Volatile Organic Compounds in Fragranced Products

Fragrance chemicals in PCHPs are not the only ones that could impact indoor air quality and are hazardous to human health. The formula components of these products that are volatile or propelled by sprays are ubiquitous and widely disseminated. The formula components essentially include water, solvents, preservative substances (parabens), fixatives (phthalates) in the case of perfumes, and surfactants in the case of cleaning products. Pressurized gases (propellants) aerosolize and deliver indoor formulation components (fragrances, acids, bases, disinfectants, organic solvents, and surfactants) that compromise the respiratory system [38].

Fragranced products developed to clean and disinfect surfaces from the SARS-Cov-19 virus, such as air sprays, hand sanitizers, and surface cleaners, emit hundreds of different VOCs and expose people to hazardous chemicals. An analysis by gas chromatography coupled to mass spectrometry (GC/MS) revealed that twenty-six fragranced "pandemic products," including the so-called "green-versions," collectively emitted approximately four hundred VOCs, of which over a hundred are potentially hazardous VOCs. In terms of concentration, limonene was the most commonly emitted VOC, which was followed by ethanol, α- and β-pinene, acetaldehyde, eucalyptol, γ-terpinene, β-myrcene, β-trans-ocimene, camphene, 3-carene, β-phellandrene, linalool, α-phellandrene, and methanol. Only a few tested virus disinfectants disclosed these hazardous VOC substances on the product labels or safety data sheets [42]. As mentioned above, even PCHPs destined to use in young children are not free from mixtures of fragrances and VOCs in their formulations. A similar analysis of GC/MS found approximately seven hundred VOCs emitted collectively from around forty baby PCHPs, of which over two hundred twenty are categorized as potentially hazardous. The predominant VOCs emitted were limonene, acetaldehyde, ethanol, α-pinene, linalool, β-myrcene, acetone, and β-pinene. Again, only a few baby products disclosed detailed descriptions of these constituents’ VOCs on the labels and safety datasheets [24]. Intriguingly about the emission of limonene is that synthetic R-limonene was more repellent than natural R-limonene in vitro tests with a mosquito assay model, while synthetic R-limonene and S-limonene displayed the same level of repellency [43]. These facts may affect indoor air quality and pose harm to humans.

Assessment of the indoor VOCs in homes as a result of spray antiperspirant deodorants and other products usage showed that the concentrations of n-butane (aerosol propellant), ethanol, acetone, and propane, exceeded the outdoor levels for over eighty percent of households studied in summer and hundred percent of homes in winter. Other emitted VOCs detected in decrescent concentrations included, for example, α-pinene, D4 siloxane, ethane, limonene, iso-pentane, toluene, xylene, isoprene, p-cymene [44]. Estimates of emitted VOCs after using water-based hand spray PCHPs indicated that the average concentrations in the indoor air of propylene glycol, 1,3-butanediol, and diethylene glycol monoethyl ether were higher than those reported in a national survey of indoor air pollution [45]. High levels of VOCs like glycol ethers were detected in indoor air owing to emissions from PCHPs (surface cleaners and air fresheners) in experiments conducted to quantify the eventual exposure of workers involved in cleaning [34]. Examination of indoor emissions of VOCs by sampling residences in Canada, China, and the U.S.A., indicated that the following highest concentration levels were limonene, toluene, m-p xylene, α-pinene, pentane, and decane (Canada and the U.S.A.), and toluene, naphthalene, ethyl-acetate, and m-p-xylene (China). In such a study, the indoor VOCs’ fluctuation and levels are linked to building microenvironments and age, the frequency of product consumption, and ventilation conditions, among other factors [46].

Interestingly, even a single application of daily-use facial in a controlled exposure to PCHPs constituents, such as ethanol, 2-propanol, benzyl alcohol, 1,3-butanediol, t-butyl alcohol, and monoterpenes, indicated that much higher concentrations of VOCs could be inhaled (ethanol ~300 times more; limonene ~16x) compared to the inhalation of typical indoor air [47].

## 4. Indoor Air Quality and Fragrance VOCs

Several studies and risk analysis reports have been conducted to assess and establish limits in the indoor air regarding the presence of fragrances and VOCs that are constituents of PCHPs. For instance, Potera [48] pointed out that “Scented Products Emit a Bouquet of VOCs”, with over a hundred VOCs found in dozens of “green”, “natural”, or “organic” PCHPs, of which more than twenty of them classified as “toxic” or “hazardous”, but none disclosed on products labels, except for one product. The most frequent VOCs found were terpenes (limonene, α- and β-pinene), ethanol, acetone, and carcinogenic chemicals like acetaldehyde, 1,4-dioxane, formaldehyde, or methylene chloride. In an experimental determination with cleaning products and air fresheners containing terpenoids and glycol ethers in a ventilated controlled 50 m^3^ room, the concentrations of d-limonene, dihydromyrcenol, linalool, linalyl acetate, and beta-citronellol emitted were several times higher (~35–180 mg/day during three days) than the air (average air concentration ~30–160 μg/m^3^) when the products were in use [34]. Glycol ethers are regulated toxic air contaminants, and terpenes can react with ozone to form secondary pollutants.

A systematic study showed that limonene and linalool were present as fragrance chemicals in 72% and 45% of the most common household product formulations analyzed worldwide. These fragrance VOCs reached concentrations of 7–140 μg/m^3^ (limonene) and 544 to 787 μg/m^3^ (linalool)from a range of 396 to 1013 μg/m^3^ of total terpenes measured during testing periods of 3 h in two different types of indoor environments [8]. Quantitative risk assessments were elaborated and issued to establish the safe levels for sensitizing fragrance materials in multiple products to assess the risk of skin sensitization to fragrance materials and limit the risk of induction of contact allergy [49]. However, the lack of data regarding the concentration–response relationships between VOC levels in civil buildings and various health issues prompted Liu and coworkers [50] to conduct a systematic review and meta-analysis. They found robust evidence for associations between increased concentrations of several VOCs and adverse health effects, mainly benzene and leukemia, asthma, and low birth weight. Another systematic review by Vardoulakis and colleagues [51] reports the negative influence of several air contaminants, including fragrance chemicals, on indoor air quality and human health. Multiple factors contribute to the decrease indoor air quality pollutants and are associated with adverse health effects, such as respiratory and cardiovascular illness, allergic symptoms, and cancers. These factors and disease outcomes are related to household characteristics, seasonal influences, and occupancy patterns. Indoor particles and VOCs (e.g., aromatic and aliphatic compounds) in the home environment are associated with increased asthma symptoms, as reviewed by Paterson and coworkers [52]. These systematic studies indicate that secondary particles that result from emitted gases and VOCs from household products decrease indoor air quality, health, and safety. Finally, a systematic study regarding the emission, indoor air concentration, and health effects in European residences indicated that out of 65 individual VOCs commonly found, over forty were from PCHPs, like air fresheners, hair sprays, deodorants, among others [53].

## 5. Discussion

Atmospheric pollution due to VOCs, mainly from fossil fuel origin, is of significant concern because they are responsible for deleterious effects on Earth’s climate and human health, like depletion of the stratospheric ozone layer and formation of tropospheric ozone, which are interconnected with the global warming due to greenhouse gases [54,55]. Food production is responsible for one-quarter of the world’s greenhouse gas emissions. Additionally, the water footprint, i.e., the volume of water used in food production, the quantity of water for direct human consumption, and the amount of food to feed billions of humans on Earth, reach exponential numbers [56]. In chemical terms, water and nutrients (food) are not the only requirements for human growth, maintenance, and welfare in the modern world. The global population demands broad categories of products to maintain personal hygiene and health. 

Consequently, the high demand for active pharmaceutical and personal care product ingredients and increased consumption of PPCPs can result in environmental contamination caused by the so-called emerging pollutants. Apart from the pharmaceutical ingredients, the fragrance industry is a multi-billionaire market providing materials for personal care and household products. Regarding the global perfume market exclusively, the projected growth of over 43 billion U.S. dollars was expected by 2028, with a compound annual growth rate of 5.0% in 2021–2028 [57]. Fragrance compounds (fragrance mixtures or essential oils) comprise formulations of specific combinations of individual materials or mixtures [58]. As fragrances and formula constituents are volatile organic compounds, consumers and workers are exposed to variable indoor concentrations of these chemicals. Fragrances and essential oils have numerous beneficial effects ranging from antisepsis [59] to aromatherapy [60]. In aromatherapy, the essential oils (volatile oils) from diverse sources, used through inhalation, local application, and skin permeation, are intended to relieve neural issues (e.g., depression, migraine, headache, insomnia), inflammatory problems (arthritis), stomach disturbances (indigestion), muscular pain, respiratory problems, among others, symptoms of health disturbances. Homeopathic therapeutists claim odorant molecules such as flavonoids or terpene activate the neural-immune axis and improve homeostasis [61]. However, even therapeutic compounds can cause adverse effects, and xenobiotics, in particular attention here, target multiple organs. Repetitive indoor exposure to fragrance chemicals poses a risk for vulnerable and sensitive persons, like asthmatic and allergic people, people who suffer from migraine, and occupational and housekeeping workers.

As aforementioned, ORs receptors are expressed in sensory neurons in olfactory systems and diverse human body tissues, exerting endocrine and regulatory functions. Ionones (α-and β-ionones, metabolic products of α-and β-pinene) acting through OR-signaling, regulation of cell cycle processes, and synthesis of pro-inflammatory mediators via HMG-reductase, possess a range of pharmacological effects: from anticancer to chemoprotection to anti-inflammatory and antimicrobial activities [15]. Despite the beneficial effects of α-and β-ionones and their role in homeostasis, they can exacerbate the aggressiveness of specific tumor types and increase the number of metastases [20]. Disruption of the endocrine system is observed for many daily use chemicals, and environmental contaminants and fragrances mixtures also contain components that can act as endocrine disruptors [22,26,28,62], posing critical concerns about their widespread uses. Fragrance constituents in PCHPs are entirely undisclosed and poorly regulated [8,16,63]. The volatilization and emission of fragrance chemicals from PCHPs depend on various factors such as frequency and quantity of use, evaporation, mass transfer, and diffusion [64]. However, fragrance chemicals are detected everywhere in several environments already investigated [5]. Thinking about these constraints and potential health risks of fragrance chemicals, the Research Institute for Fragrance Materials, Inc. (RIFM) has committed to the risk assessment and evaluation of safety data for fragrance materials through gathering data, estimating exposure, and assessing safety, and publishing and distributing scientific information regarding fragrance substances [58].

## 6. Conclusions

The human population reached 8 billion individuals by the end of 2020. The food and energy industries are not the sole potential generators of environmental contaminants. The demand for pharmaceuticals, personal care, and household products for a better quality of life proportionally involves the formulation of thousands of chemical ingredients. Formula components of PCHPs entering various environmental compartments comprise “emerging contaminants of concern”. Fragrance mixtures are attractive and vital components of PCHPs that sensibilize the olfactory system and improve the sensorial perception of cosmetics and household products. Despite the beneficial effects of fragrances and essential oils, they can harm human health, particularly in the sensitive part of populations. Fragrance and some formula constituents are VOCs that, according to the frequency, the quantity of use, and evaporation and diffusion, can impact indoor air quality.

As a take-home message regarding fragrance chemicals in PCHPs, even if most fragrance chemicals are assigned low risk through risk assessment and safety evaluation, secondary pollutants that negatively affect human health can emerge. In addition, hundreds of undisclosed fragrance-related chemicals in product formulations of PCHPs can trigger or intensify episodic and chronic symptoms of allergies, headaches, and cardiovascular diseases in sensitive organisms. In worse cases, fragrance chemicals interfere with the neuroendocrine-immune axis promoting cancer and developmental problems. Constant surveillance and analysis of fragrance chemicals in PCHPs and their formula components are critical.

## 7. Materials and Methods

This systematic review followed the PRISMA (Preferred Reporting Items for Systematic Reviews and Meta-Analysis) guidelines [65]. The PubMed, ScienceDirect, and Google Scholar electronic databases were searched. The interrogation of databases was without the restriction of the date of publication. The search terms included: “synthetic fragrances”, “synthetic fragrances and adverse health effects”, “synthetic fragrances and indoor air quality”, “odorant VOCs”, “VOCs and indoor air quality”, “Indoor VOCs and adverse health effects”. The selected literature included reviews and original research articles. Thirty-six original articles were initially selected from the databases using the primary keywords and adherence to the reviewed topics. Additional articles from the literature were selected to expand the concepts regarding the field, and references were further complemented with essential information on the topics covered in this review.

## Figures and Tables

**Table 1:** Examples of fragrance chemicals and PHCPs’ formula components associated with adverse health effects.

Fragrance Chemicals and Formula Components	Implication on Human Health	Ref.
Phthalates, synthetic musks, and terpenes (e.g., citral, linalool, limonene, geraniol, citronellol, eugenol, farnesol)	Neurotoxicity and neural issues dizziness, seizures, loss of coordination, depression, migraine, headache, insomnia	[16,25]
Fragrance-related phthalates and synthetic musks	Endocrine disruption Reproductive and sexual abnormalities issues	[22,27,28]
Terpenes, saturated and unsaturated aldehydes, and acetals	Allergies (cutaneous and pulmonary hypersensitivity) allergic reactions (fragrance allergy), contact dermatitis, contact reactions (contact urticaria), photosensitivity, and respiratory disorders	[30]
Terpenes, VOCs (aromatic and aliphatic compounds)	Respiratory issues Asthma, difficulty breathing, coughing, shortness of breath	[38]
Ultrafine particles	Cardiac symptoms Increased risk of cardiovascular health hazards and cardiac insufficiency	[39]
Lactones (e.g., mintlactone) and phthalates	Mutagenesis and cancer	[39,40]
VOCs (diverse classes)	Autoimmune diseases, including pulmonary Disease, atherosclerosis, and rheumatoid arthritis	[41]

## Data Availability

Not applicable.
